# Supplementary figures and images for: When does selection favor learning from the old? Social learning in age-structured populations
Source: PLoS One. 2022 Apr 15;17(4):e0267204. doi: 10.1371/journal.pone.0267204 (PMC9012401; doi:10.1371/journal.pone.0267204)

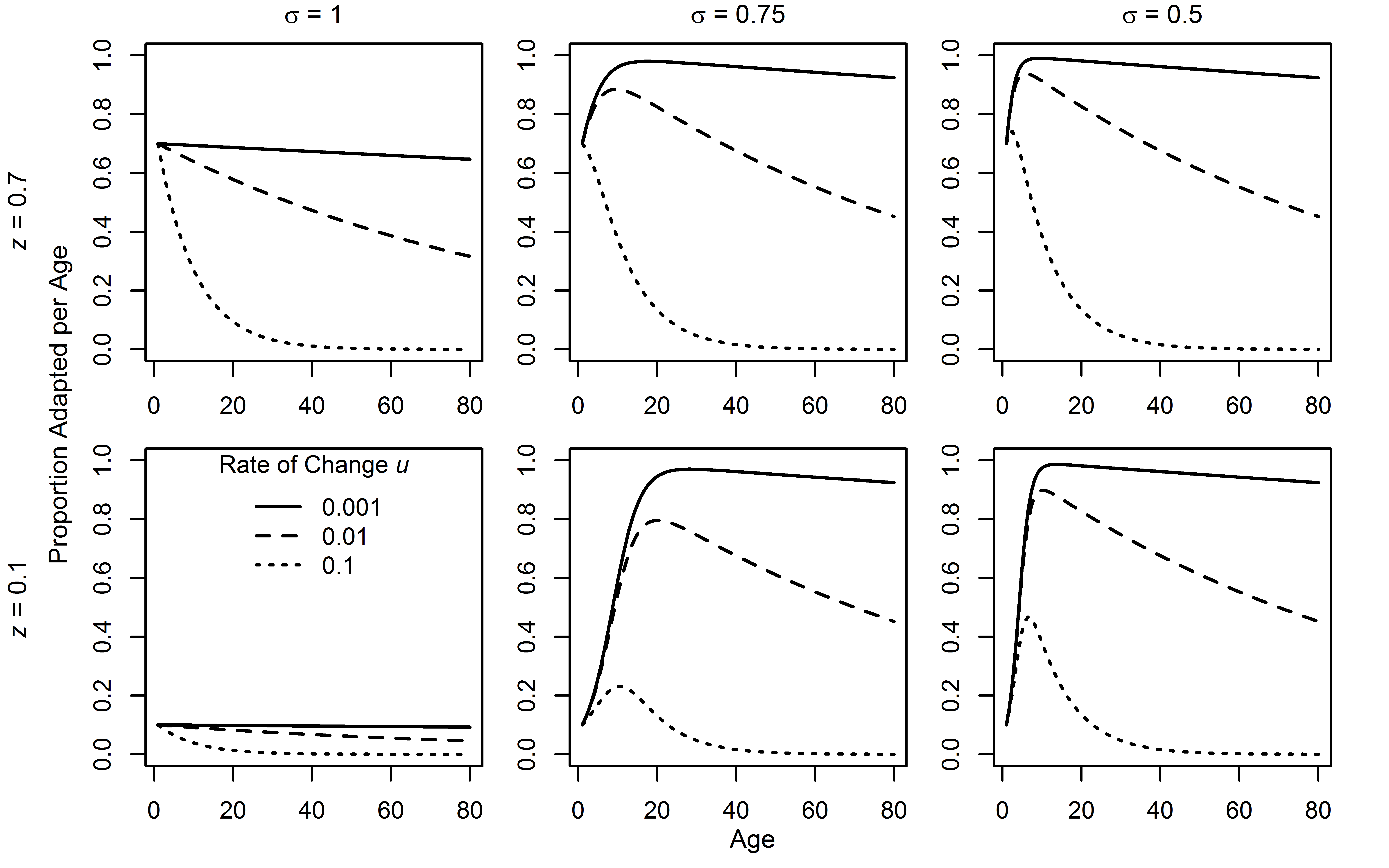

Supplement: S1 Fig — Plots show results for different values of z (top row: z = 0.7, bottom row: z = 0.1), σ (left: σ = 1, center: σ = 0.75, right: σ = 0.5) and u (solid: u = 0.001, dashed: u = 0.01, dotted: u = 0.1.). (TIFF) [file pone.0267204.s001.tiff]

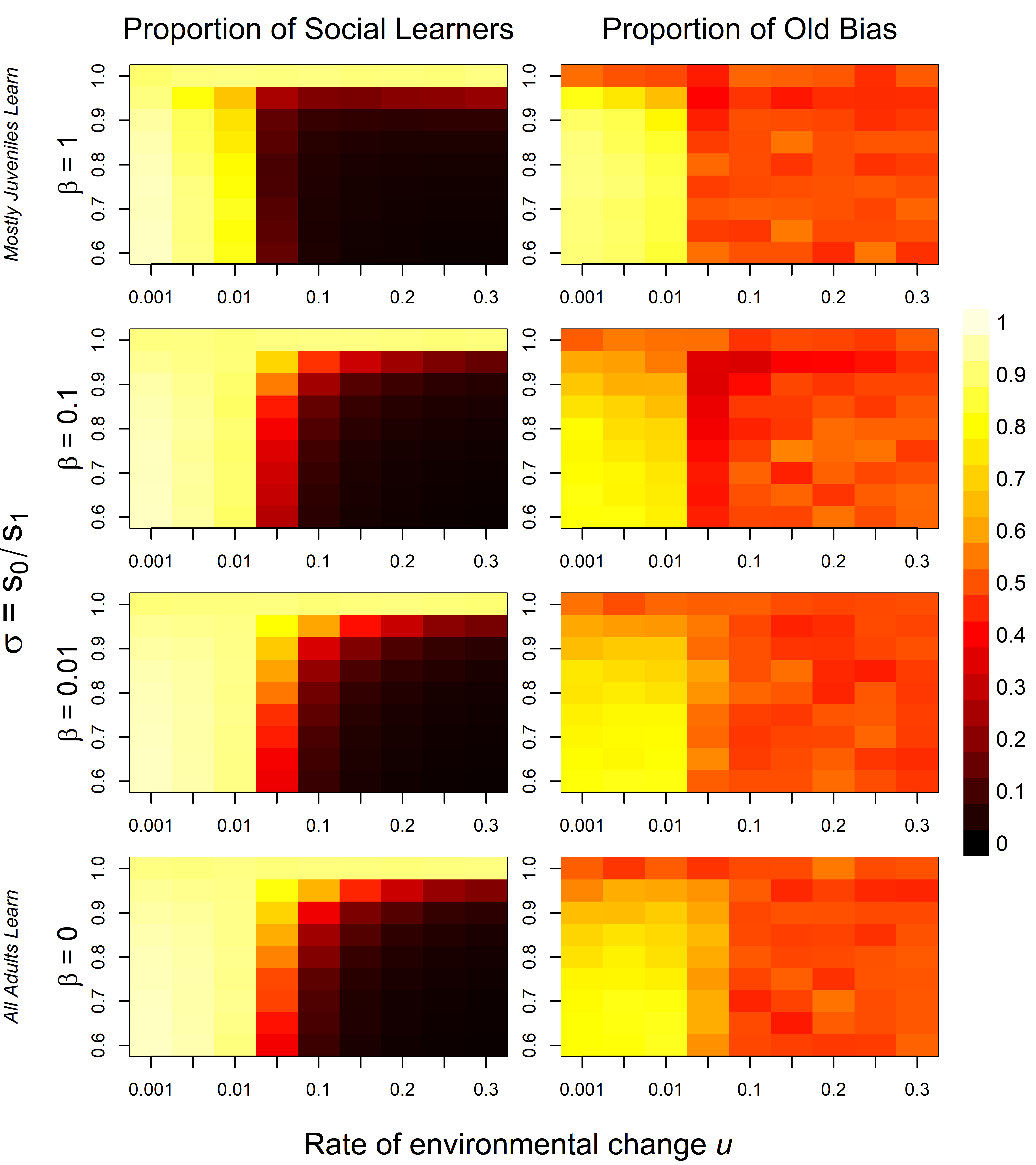

Supplement: S3 Fig — Proportion of social learners (left) and old bias (right) for temporal individual-based model with adult learning and social learning error (ϵ = 0.3). Rows show results for different age-related exponential decline rates in learning; β = 1 means mostly juveniles learn, β = 0 means all age classes are equally able to learn. Squares represent different parameter combinations for the rate of environmental change u and the strength of viability selection σ. Lighter colors indicate higher proportions, darker colors lower proportions. Results are averaged over the last 5000 time steps of 10 independent 7000 time-step simulations per parameter combination. Other parameter values are: c = 0.05, z = 0.5, s1 = 0.9, μ = 0.005. (TIFF) [file pone.0267204.s003.tiff]
