# Supplementary material for: When does selection favor learning from the old? Social learning in age-structured populations
Source: PLoS One. 2022 Apr 15;17(4):e0267204. doi: 10.1371/journal.pone.0267204 (PMC9012401; doi:10.1371/journal.pone.0267204)
Supplement: Attachment — Submitted filename: review.pdf [file pone.0267204.s004.pdf]

Dear Editor, dear authors,

This article entitled "When does selection favor learning from the old? Social learning in age-structured populations" models the evolution of learning strategies in an age-structured population and seeks to establish the conditions under which it is adaptive to learn preferentially from the old rather than the young.

The article is well written. The model is rigorous. The analyses are accurate. It is certainly an article that deserves to be published in PloS ONE.

However, I have one rather substantial criticism that I believe requires a significant correction to the article.

The most significant assumption of the model, which is very little discussed and yet has very important consequences, is the assumption that *adults cannot learn*.

As a result of this assumption, the only reason why old people may have better information than young people is because they have been more “tested” by mortality (demographic filtering).

However, this is only the case *in this model*, owing to the specific assumption that adults cannot learn.

In real life, demographic filtering is very unlikely to be the most general reason why old people may be better sources of information than young people. Old people are rather a better source of information because they have more *experience*. Having more experience has nothing to do with having survived longer. Having more experience is about having had more opportunities to learn, i.e. to have received more feedbacks from the environment. But this cannot be captured in a model where adults cannot learn. So the model fails to capture what is probably the most important and general reason why the old have better information than the young.

In itself, this is not a problem. It’s perfectly fine to build simplified models that capture only a part of reality.

However, the problem is that the authors do not acknowledge this limitation. In particular, they purport to relate their modeling results to empirical observations (lines 381-395), even though these observations are in fact very unlikely to be the consequence of demographic filtering, but much more likely to be consequence of differential experience. For example, if 15-month-olds are more likely to copy behaviors performed by an adult versus a two-year-old child (as cited by the authors, line 382), it is very likely NOT because adults have been tested by mortality, but more simply because they have had more opportunities to learn. And this is probably the same for all the empirical data cited by the author (lines 381-395).

So the model just *superficially* looks like it predicts empirical and real life observations while, in reality, it fails to capture the likely true cause of these observations (differential experience). The authors must make this point explicit. Otherwise they misled the readers into believing that they are reading a model that is much more interesting than it really is.

For example, the authors are extremely misleading in line 192 after their paragraph on empirical studies: "Taken together, these studies seem to imply that under most circumstances learners preferentially attend to older, more experienced, individuals. Our modeling results show that this can be a good strategy, but only when the environment is relatively stable, adaptive behavior is hard to acquire and confers large survival advantages." They present empirical observations that are caused by differential experience and claim that they are explained by their model that only captures demographic filtering!

In the article, the authors express their view on the right way to do modeling (line 334-337) a point on which I agree with them. Let me then add a short patronizing lesson. The aim of modeling is not to reproduce patterns that seem to resemble reality, without asking oneself whether the mechanism at the origin of these patterns is also in line with reality. Otherwise, modeling is nothing more than a magic trick. In a useful model, both the pattern produced AND the mechanism causing it must be in line with empirical data.

Small comments:

1)

Regarding "age structure and Rogers' paradox". In the abstract, in the discussion, and also in the title of this section, the authors suggest that there is a real scientific question: "Does adding age structure resolve Rogers' paradox?".

This is rather artificial. There is nothing surprising about the fact that age structure does not resolve Rogers' paradox in the absence of a preferential transmission of accurate information. The authors should rather say something like: ”We replicate Rogers' paradox with age structure". That would be less misleading.

2) This sentence is not clear at all (line 359): “Even though social learning did not increase mean population fitness, in combination with demographic filtering, it enabled the population to reach much higher adaptation levels.”
