# Supplementary material for: When does selection favor learning from the old? Social learning in age-structured populations
Source: PLoS One. 2022 Apr 15;17(4):e0267204. doi: 10.1371/journal.pone.0267204 (PMC9012401; doi:10.1371/journal.pone.0267204)
Supplement: Attachment — Submitted filename: Response to Reviewers.docx [file pone.0267204.s005.docx]

Dear author,

Thank you for submitting this serious and solid paper to PLOS ONE. After a delay due to difficulties in finding reviewers, I have now received two reviewer reports, both positive overall. The only substantial critique is raised by Reviewer 1's report. Please address it as fully as possible. If I am satisfied with the way this point is addressed I might accept the paper directly and not send it back.

(Also, one small thing: please format the references section following standard guidelines and reread it carefully, right now the references are neither numbered nor ordered and there are typos, e.g. geocentracy for gerontocracy.)

Again, thank you for submitting your work to our journal.

Sincerely,

Olivier Morin

Dear Dr. Morin,

We would like to thank you and the reviewers for the thorough evaluation of our manuscript. We hope that in this revised version we have addressed all reviewer comments and suggestions and are convinced that this revision further improved the manuscript and highlighted the contributions of our model.

With respect to the main issue raised by reviewer 1, we now clarify that the agreement with empirical results could be due to missing factors such as later learning and experience differences. In humans, where many skills take decades to develop, experience might very well be the main reason to copy older individuals as we now discuss in more detail. However, as stated in the introduction, the goal of this model was to examine exactly two factors, namely the influence of environmental change and the demographic filtering of adaptive information in older age classes through viability selection. As this literature on the interplay between social learning and demography is still in its infancy, we focus on the simplest scenarios.

However, to investigate how robust our results are and if they really only depend on the assumption that adults do not learn as claimed by the reviewer, we now extend our individual-based simulations to scenarios where adults also learn with some (exponentially declining) probability (shown in Fig. 6 and S3 in the supplementary material). This analysis reveals that our major results do NOT depend on the assumption that only juveniles learn, but on the assumption that juveniles learn to a somewhat greater degree, which is likely to be the case in most biological systems. In fact, even if all age classes equally update their behavior, our results with copying error stay the same and, without copying error, there is only a small region in parameter space that favors a pronounced “copy-the-old” bias.

We have also re-formatted the reference section and apologize for any inconvenience this might have caused.

To aid orientation, reviewer comments are in black, our responses are in blue and new text added to the manuscript is in green.

Sincerely,

Dominik Deffner and Richard McElreath

Reviewer #1: Dear Editor, dear authors,

This article entitled "When does selection favor learning from the old? Social learning in age-structured populations" models the evolution of learning strategies in an age-structured population and seeks to establish the conditions under which it is adaptive to learn preferentially from the old rather than the young.

The article is well written. The model is rigorous. The analyses are accurate. It is certainly an article that deserves to be published in PloS ONE.

However, I have one rather substantial criticism that I believe requires a significant correction to the article.

The most significant assumption of the model, which is very little discussed and yet has very important consequences, is the assumption that adults cannot learn.

As a result of this assumption, the only reason why old people may have better information than young people is because they have been more “tested” by mortality (demographic filtering).

However, this is only the case in this model, owing to the specific assumption that adults cannot learn.

In real life, demographic filtering is very unlikely to be the most general reason why old people may be better sources of information than young people. Old people are rather a better source of information because they have more experience. Having more experience has nothing to do with having survived longer. Having more experience is about having had more opportunities to learn, i.e. to have received more feedbacks from the environment. But this cannot be captured in a model where adults cannot learn. So the model fails to capture what is probably the most important and general reason why the old have better information than the young.

In itself, this is not a problem. It’s perfectly fine to build simplified models that capture only a part of reality.

However, the problem is that the authors do not acknowledge this limitation. In particular, they purport to relate their modeling results to empirical observations (lines 381-395), even though these observations are in fact very unlikely to be the consequence of demographic filtering, but much more likely to be consequence of differential experience. For example, if 15-month-olds are more likely to copy behaviors performed by an adult versus a two-year-old child (as cited by the authors, line 382), it is very likely NOT because adults have been tested by mortality, but more simply because they have had more opportunities to learn. And this is probably the same for all the empirical data cited by the author (lines 381-395).

So the model just superficially looks like it predicts empirical and real life observations while, in reality, it fails to capture the likely true cause of these observations (differential experience). The authors must make this point explicit. Otherwise they misled the readers into believing that they are reading a model that is much more interesting than it really is.

For example, the authors are extremely misleading in line 192 after their paragraph on empirical studies: "Taken together, these studies seem to imply that under most circumstances learners preferentially attend to older, more experienced, individuals. Our modeling results show that this can be a good strategy, but only when the environment is relatively stable, adaptive behavior is hard to acquire and confers large survival advantages." They present empirical observations that are caused by differential experience and claim that they are explained by their model that only captures demographic filtering!

In the article, the authors express their view on the right way to do modeling (line 334-337) a point on which I agree with them. Let me then add a short patronizing lesson. The aim of modeling is not to reproduce patterns that seem to resemble reality, without asking oneself whether the mechanism at the origin of these patterns is also in line with reality. Otherwise, modeling is nothing more than a magic trick. In a useful model, both the pattern produced AND the mechanism causing it must be in line with empirical data.

Thank you for this important point. We now clarify that the agreement with empirical results could be because of missing factors such as later learning and experience differences. This is especially true for cumulative and skill-intensive human culture:

“However, we note that any agreement with empirical results could of course also be due to missing factors. Many essential skills in real animals, and humans in particular, take generations to evolve and years to develop, which is particularly true for cumulative and causally opaque human culture (Koster et al., 2020; Deffner and McElreath, 2020). If adaptive behaviour takes time and practice to develop, older individuals may be more likely to have mastered high skill levels and may therefore—not because of demographic filtering—be preferable cultural role models.” (lines 420-426)

However, we disagree with the claim that our entire result depends on the strict assumption that only juveniles learn and repeated learning opportunities will uniformly lead to preferential learning from the old. We now include simulation results where adults also learn with a certain age-dependent probability (Fig. 6 and S3 in the supplementary material). These simulations show that our major results do NOT depend on the assumption that only juveniles learn, but on the assumption that juveniles learn to a somewhat greater degree, which is likely to be the case in most biological systems. In fact, even if all age classes equally update their behavior, our results with copying error stay the same and, without copying error, there is only a small region in parameter space that favors a pronounced “copy-the-old” bias.

“Finally, to better understand the influence of the assumption that only juveniles learn and to determine the generality of our findings, we run additional individual-based simulations with temporally changing environments and now also let adults update their behavior with a certain age-dependent probability. Specifically, we assume that the probability to learn (through either individual or social learning) declines exponentially, with parameter β giving the rate of age-related decline: e−β(Age−1). This formulation implies that juveniles (Age = 1) always learn and adults become less likely to update their behavior as they become older. Figure 6 shows proportions of social learners (left) and old bias (right) for different age-related exponential decline rates in learning. If juveniles are substantially more likely to learn compared to older individuals (top rows), the results replicate previous findings where only juveniles could learn. As more older adults learn each time step and, thus, are less likely to become outdated, social learning becomes more prevalent in the population and learners progressively shift towards older versus younger role models. However, even if all adults learn each time step (bottom row), there is only a small region in parameter space—marked by moderate environmental stability and strong viability selection—that favors a pronounced “copy-the-old” bias even though older individuals have had more learning opportunities. Moreover, Figure S3 in the supplementary material reveals that adult learning does not qualitatively change results for analyses with social learning errors. Overall, these analyses show that our results do not depend on a specific assumption about exclusively juvenile learning, but on the biologically plausible assumption that adults become less likely to change their behavior as they age.” (lines 329-349)

Small comments:

1) Regarding "age structure and Rogers' paradox". In the abstract, in the discussion, and also in the title of this section, the authors suggest that there is a real scientific question: "Does adding age structure resolve Rogers' paradox?".

This is rather artificial. There is nothing surprising about the fact that age structure does not resolve Rogers' paradox in the absence of a preferential transmission of accurate information. The authors should rather say something like: ”We replicate Rogers' paradox with age structure". That would be less misleading.

Thank you for the comment. We agree that, in hindsight, there is probably nothing surprising about this finding, as social learning still does not increase mean fitness of individual learners, which is a prerequisite for Rogers’ paradox to disappear. However, based on previous findings that show that spatial population structure can solve the paradox (Rendell, Fogarty & Laland, 2010), many researchers might have been led to believe (at least we were) that age structure might have a similar effect. Age structure and viability selection introduce a second adaptive force in addition to individual learning that might have been expected to increase population fitness in a similar way as it increases the proportion of adapted individuals in the population (as shown in Fig.2). That this is not the case is a new theoretical result and not a replication of the original paradox.

2) This sentence is not clear at all (line 359): “Even though social learning did not increase mean population fitness, in combination with demographic filtering, it enabled the population to reach much higher adaptation levels.”

Thanks. We have now rewritten this sentence to make it clearer:

“Even though social learning did not increase mean population fitness, in combination with demographic filtering, it enabled the population to reach much higher proportions of adapted individuals.” (line 383)

Reviewer #2: First, I should say that I have no major critiques of the manuscript and I would be happy to see it published as-is. Both the mathematical and agent-based models are as simple as possible given the research questions, and the interpretation of their output appears to be sound. I only have a few minor recommendations:

Thank you very much for the kind words and positive evaluation of our work.

- As far as I can tell "demographic filtering" is not defined in the paper, but appears throughout. It is worth defining this for readers (like myself) who are unfamiliar with the term.

Thanks for this suggestion. The term is now defined where it is first introduced:

“The differential survival of adapted individuals to high ages (what we call “demographic filtering”), however, can lead to higher frequencies of social learning and cumulative improvements in adaptation levels.” (lines 61-63)

- Part of the aims statement, on lines 56-58, is quite broad and hard to follow (for me, and possibly for other readers). It reads: "How do demographic and cultural forces interact in adaptation and population growth and what are the minimal conditions for such forces to make it worthwhile to copy older individuals?" I would consider making the "demographic and cultural forces interact in adaptation and population growth" part more specific, because right now I could think of many possible research questions that fit that description.

Thank you. We have now changed this to:

“How does demographic population structure and social learning interact in adaptation and population growth” (lines 57-59)

- It might be worth clarifying the description of Figure 1 on lines 160-162. I think the fact that the black shapes and colored lines represent two different sets of results is confusing on an intuitive level since they are in the same position (which I know is the point being made, but still might be worth clarifying).

Thanks for the suggestion. We have now rewritten the description of Figure 1:

“Figures 1C,D show fitness for populations with only individual learners (black shapes) and populations with individual and social learners (colored lines). This comparison reveals that fitness in both models is solely determined by the fitness of individual learners and does not change with the introduction of social learners.” (lines 162-163)

- Have you considered including Figure S3 in the body text? I think it does an excellent job of communicating the influence of environmental stability, and would enhance readers' understanding of that result.

Thanks, we agree this figure adds interesting detail that can enhance readers’ understanding. We have now included it as Fig. 5 in the main text.
